# Glaucoma-causing *ADAMTS17* mutations are also reproducibly associated with height in two domestic dog breeds: selection for short stature may have contributed to increased prevalence of glaucoma

**DOI:** 10.1186/s40575-019-0071-6

**Published:** 2019-05-17

**Authors:** Emily C. Jeanes, James A. C. Oliver, Sally L. Ricketts, David J. Gould, Cathryn S. Mellersh

**Affiliations:** 1 Centre for Small Animal Studies, Animal Health Trust, Lanwades Park, Kentford, Newmarket, CB8 7UU UK; 2 Canine Genetics Research Group, Kennel Club Genetics Centre, Animal Health Trust, Lanwades Park, Kentford, Newmarket, CB8 7UU UK; 3 Davies Veterinary Specialists, Manor Farm Business Park, Higham Gobion, Hitchin, SG5 3HR UK

**Keywords:** *ADAMTS17*, Height, Primary open angle Glaucoma, Primary Lens luxation, Weill-Marchesani-like syndrome

## Abstract

**Background:**

In humans, *ADAMTS17* mutations are known to cause Weill-Marchesani-like syndrome, which is characterised by lenticular myopia, ectopia lentis, glaucoma, spherophakia, and short stature. Breed-specific homozygous mutations in *ADAMTS17* are associated with primary open angle glaucoma (POAG) in several dog breeds, including the Petit Basset Griffon Vendeen (PBGV) and Shar Pei (SP). We hypothesised that these mutations are associated with short stature in these breeds.

**Methods:**

Two hundred thirty-three PBGV and 66 SP were genotyped for their breed-specific *ADAMTS17* mutations. The height of each dog was measured at the withers. We used linear (per allele) regression to assess the association between *ADAMTS17* mutations and height as a continuous variable, and linear regression and likelihood ratio tests to assess the shape of the association by comparing a general model with a linear (per allele) model.

**Results:**

The adjusted mean heights of affected, carrier, and clear PBGV were 33.49 cm (*n* = 21, 95% CI 32.78–34.19 cm), 34.88 cm (*n* = 85, 95% CI 34.53–35.25 cm), and 34.92 cm (*n* = 121, 95% CI 34.62–35.21 cm), respectively. The mean heights of affected, carrier, and clear SP were 43.96 cm (*n* = 9, 95% CI 41.88–46.03 cm), 47.56 cm (*n* = 28, 95% CI 45.50–48.63 cm), and 48.95 cm (*n* = 23, 95% CI 47.80–50.11 cm), respectively. There was a significant difference between the height of affected and clear animals in the PBGV (*P* = 0.001) and the SP (*P* = < 0.0001).

**Conclusions:**

*ADAMTS17* POAG mutations are significantly associated with height in these breeds.

**Electronic supplementary material:**

The online version of this article (10.1186/s40575-019-0071-6) contains supplementary material, which is available to authorized users.

## Plain English summary

Mutations in the *ADAMTS17* gene in humans cause Weill-Marchesani-like syndrome, which is a connective tissue disorder. Individuals that carry two mutated copies of the gene are affected; if an individual is heterozygous for a specific mutation they are not clinically affected, but can pass the mutation on to their offspring. Their offspring could also be affected, if they inherit the mutation from both parents. Affected individuals are short in stature, with undersized fingers and toes, and also suffer from eye problems including abnormal and unstable lenses, short-sightedness and secondary glaucoma. These changes are caused because the *ADAMTS17* gene is involved in the development of connective tissues of the limbs and eyes.

Mutations in *ADAMTS17* have also been identified in several dog breeds and are associated with inherited glaucoma and unstable lenses. Association of these mutations with height in dogs has not previously been investigated. We hypothesised that dogs carrying *ADAMTS17* mutations would be shorter than dogs that are homozygous for the corresponding wildtype alleles, as it is likely that *ADAMTS17* is important for correct development of the limbs in dogs as well.

The DNA of PBGV and SP was tested to assess whether they were genetically affected (i.e. were homozygous for their breed-specific mutation), were carriers (i.e. were heterozygous for their breed-specific mutation), or were clear of the mutation (i.e. were homozygous for the wildtype allele). The height of these dogs was measured at the withers (shoulder blades), and the heights of affected, carrier and clear dogs were compared in both breeds.

An association between *ADAMTS17* mutations and height was found, with affected individuals being significantly shorter on average than both carriers of the mutation and clear dogs. Our interpretation of these data is that selecting dogs to be of a specific short height may have increased the frequency of the mutations within these breeds.

## Background

Glaucoma is the term used to describe a group of diseases which result in the progressive death of retinal ganglion cells (RGCs) and their axons [1]. Several forms of this disease exist in the dog, but a common feature is an elevated intraocular pressure (IOP) which results in compression and insult to the RGCs [2]. Canine glaucomas are all considered to be due to an abnormality in aqueous humour flow or drainage, rather than due to abnormalities in aqueous humour production [1]. Glaucoma which develops without an antecedent ocular disease (such as uveitis or trauma) is classified as primary glaucoma [1]. Different subclasses of primary glaucoma exist, but they are all known or suspected to be hereditary [1]. Causal genetic mutations have been identified for hereditary primary glaucoma in many canine breeds [3–8].

POAG is a common condition in the Petit Basset Griffon Vendeen (PBGV), with an incidence of 10.4% within the breed [9]. Affected individuals may also present with lens luxation as part of the condition [9]. The causal mutation is a 4.96 Mb inversion with a break point disrupting the *ADAMTS17* gene on canine chromosome 3 at 45,768,123 base pairs (CanFam 3.1); with the other breakpoint being in a downstream intergenic region, and it is inherited in an autosomal recessive manner [3]. *ADAMTS17* is part of the *ADAMTS* family of genes which are involved in the maintenance and turnover of the extracellular matrix (ECM) [10].

A different mutation in *ADAMTS17* (a deletion of six base pairs on canine chromosome 3 at co-ordinates 40,935,387 – 40,935,393 (CanFam 3.1) has more recently been identified in the Shar Pei (SP) [5]. As in the PBGV, this mutation causes an autosomal recessive form of POAG, and primary lens luxation (PLL) is also associated with this mutation [5, 11]. An independent *ADAMTS17* mutation has been shown to cause PLL in several breeds [12, 13]. Gould et al. [13] showed that the frequency of this c.1473 + 1 G > A *ADAMTS17* mutation, that segregates in multiple breeds, ranged from 0.02 to 0.39, leading to speculation that positive selection for this mutation could exist. Mutations in other *ADAMTS* genes have also been shown to cause POAG in other breeds [4, 6–8].

The *ADAMTS* family of genes is known to be essential for correct development of the ECM elsewhere in the body. Mutations in *ADAMTS17* in humans cause Weill-Marchesani-like syndrome (WMLS) [14]. Consistent with POAG in the PBGV and SP, this is an autosomal recessive condition. WMLS is a rare connective tissue disorder characterised by lens luxation, microspherophakia, myopia, and glaucoma, and also short stature [14]. *ADAMTS17* may regulate the composition of fibrillin isoforms [15]. It is expressed during mouse embryonic development in the lens zonules and trabecular meshwork of the eye and in the perichondrium of developing long bones, as well as in other organs, influencing development of the ECM in these areas [15]. It is likely that *ADAMTS17* plays a similar role in the dog.

The majority of the breeds in which *ADAMTS* mutations have been described are short in height. We hypothesised that dogs homozygous for *ADAMTS17* mutations would be shorter than heterozygous and wild type dogs. An association between height and POAG genotype would be consistent with our hypothesis that selection for short height has resulted in *ADAMTS17* mutations reaching high frequencies within some purebred dog populations.

## Results

### PBGV results

#### Population data

Height data from 233 PBGV were collected. Of these, 21 (9%) were affected, 87 (37%) were carriers and 125 (54%) were clear.

Data on biological sex were available for all of the 233 PBGV in the study, of which 93 (40%) were male and 140 (60%) were female. Significantly more female than male dogs were presented for examination (*P* = < 0.01). The sex distribution was not significantly different between genotype groups (*P* = > 0.05) (Table 1). Height was significantly different between males and females (*P* = < 0.0001). The mean height of females was 34.19 cm (SD 1.88) and the mean height of males was 35.63 cm (SD 1.63).

**Table 1:** Sex distribution of PBGV, with comparison between genotypes

	Total	Affected	Carrier	Clear
Female	140 (60.09%)	13 (61.90%)	54 (62.07%)	73 (58.40%)
Male	93 (39.91%)	8 (38.09%)	33 (37.93%)	52 (41.60%)
Total	233	21	87	125

Data on date of birth were available for 227 of the 233 PBGV in the study (Table 2). The Kruskal-Wallis one-way ANOVA revealed that there was a significant difference in the median ages between genotypes (*P* = 0.002). Mann-Whitney U tests with Bonferroni correction revealed that there was a statistically significant difference in the age between affected and clear PBGV (*P* = 0.006), but not between carrier and clear PBGV (*P* = 0.04), and affected and carrier PBGV (*P* = 0.35).

**Table 2:** Age of PBGV, with comparison between genotypes

	Median Age	Number of dogs	IQR
Total	4.46 years	*n* = 227	4.73 years
Affected	5.31 years	*n* = 21	3.93 years
Carrier	4.96 years	*n* = 85	4.62 years
Clear	3.68 years	*n* = 121	4.27 years

### Comparisons between measuring techniques

We assessed the correlation between the stick and laser height measuring technique for 13 dogs that had been measured using both (Table 3). The correlation was 0.58, indicating the two are correlated, albeit weakly (*P* = 0.04). Dogs were typically estimated to be shorter when measured with the laser method than with the stick measure. There was a significant difference between the means (*P* = 0.0006).

**Table 3:** Comparison of height measurement results between the stick measurement technique and the laser measurement technique

	Mean Height	Number of dogs	SD
Height (stick measurement)	34.35 cm	13	1.78
Height (laser measurement)	32.42 cm	13	1.34

It was not possible to assess the correlation between measurements of PBGV taken by the authors with measurements taken by owners, because no PBGV were measured by both the authors and their owners; we only requested owners to measure their PBGV if we could not measure the PBGV ourselves for logistical reasons.

The correlation between repeat measures using the stick was 0.79 in the seven dogs that had been measured twice (*P* = 0.03) (Table 4). The difference between the means was not statistically significant (*P* = 0.07).

**Table 4:** Comparison of height measurement results between repeated measurements using the stick measurement technique

	Mean Height	Number of dogs	SD
Height (first measurement)	34.43 cm	7	1.10
Height (repeat measurement)	35.00 cm	7	0.91

#### Height data

The mean height of affected PBGV (*n* = 21) was 33.41 cm (SD 2.67). The mean height of carrier PBGV (*n* = 87) was 34.79 cm (SD 1.85). The mean height of clear PBGV (*n* = 125) was 34.98 cm (SD 1.73).

Using Pearson’s correlation, there was no correlation between height and age for the 227 PBGV for which the date of birth was available (*P* = 0.26) (Table 5). Linear regression also confirmed this.

**Table 5:** Comparison of height and age of PBGV

Age group	Number of dogs	Median Height	IQR
Quartile 1	*n* = 57	35.00 cm	2.00 cm
Quartile 2	*n* = 56	34.40 cm	2.68 cm
Quartile 3	*n* = 57	35.00 cm	2.54 cm
Quartile 4	*n* = 57	35.00 cm	2.50 cm

**Fig. 1:**
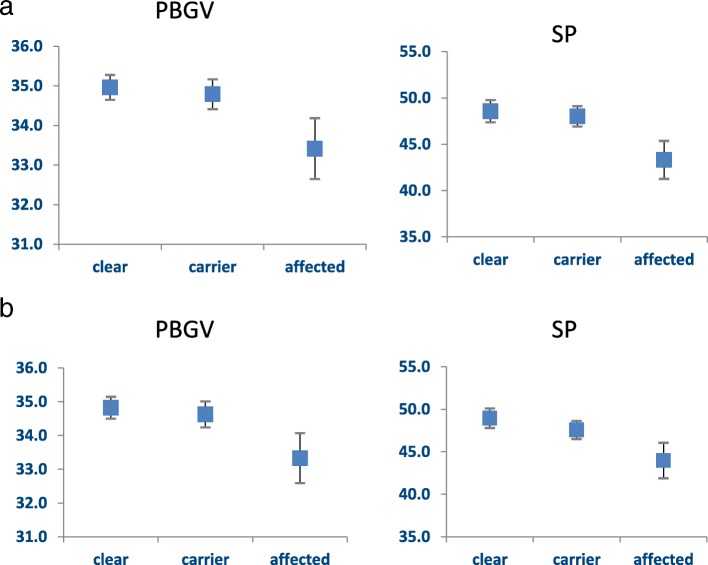
Association between height and *ADAMTS17* genotype. **a**. Unadjusted mean heights and 95% confidence intervals for each genotypic category. **b**. Age and sex-adjusted mean heights and 95% confidence intervals for each genotypic category

Unadjusted linear regression revealed a statistical association between genotype and height (*P* = 0.006), and the association appeared linear, although only marginally (*P*-value for departure from linearity = 0.06) (Fig. 1a).

We next conducted an analysis of height and genotype comprising height using all measures that adjusted for sex, age and method of measurement. The association reduced in significance (*P* = 0.02). With adjustment for the confounding effects of these variables, the association now appeared to depart from linearity (*P*-value = 0.02) (Fig. 1b). Adjusted mean heights of affected PBGV (*n* = 21) was 33.49 cm (95% CI 32.78–34.19). The mean height of carrier PBGV (*n* = 85) was 34.88 cm (95% CI 34.53–35.23). The mean height of clear PBGV (*n* = 121) was 34.92 cm (95% CI 34.62–35.21). Using a genotypic model with adjustment as above, there was a significant difference in height between clear and affected PBGV (*P* = 0.001) but not between clear and carrier PBGV (*P* = 0.91).

### SP results

#### Population data

Height data from 66 SP were collected. Of these, 9 (14%) were affected, 31 (47%) were carrier and 26 (39%) were clear.

Data on biological sex were available for all 66 SP in the study, of which 22 (33%) were male and 44 (67%) were female. Significantly more female than male dogs were presented for examination (*P* = < 0.01). The sex distribution was not significantly different between genotype groups (*P* = > 0.05) (Table 6). Height was significantly different between males and females (*P* = < 0.0001). The mean height of females was 46.40 cm (SD 3.07) and the mean height of males was 50.00 cm (SD 3.04).

**Table 6:** Sex distribution of SP with comparison between genotypes

	Total	Affected	Carrier	Clear
Female	44 (66.67%)	8 (88.89%)	18 (58.06%)	18 (69.23%)
Male	22 (33.33%)	1 (11.11%)	13 (41.94%)	8 (30.77%)
Total	66	9	31	26

Data on date of birth were available for 60 of the 66 SP in the study (Table 7). The Kruskal-Wallis one-way ANOVA revealed that there was a significant difference in the ages between genotypes (*P* = 0.007). Mann-Whitney U tests with Bonferroni correction revealed that there was a statistically significant difference in the age between affected and clear SP (*P* = 0.002), affected and carrier SP (*P* = 0.004), but not between carrier and clear SP (*P* = 0.64).

**Table 7:** Age of SP with comparison between genotypes

	Median Age	Number of dogs	IQR
Total	3.33 years	*n* = 60	2.70 years
Affected	5.80 years	*n* = 9	3.83 years
Carrier	2.51 years	*n* = 28	2.76 years
Clear	2.98 years	*n* = 23	2.01 years

### Comparisons between measuring techniques

All SP were measured either with the commercially available measuring stick, or by using a spirit level to mark the height of the withers on a wall then measuring the distance between the mark and the floor.

**Fig. 2:**
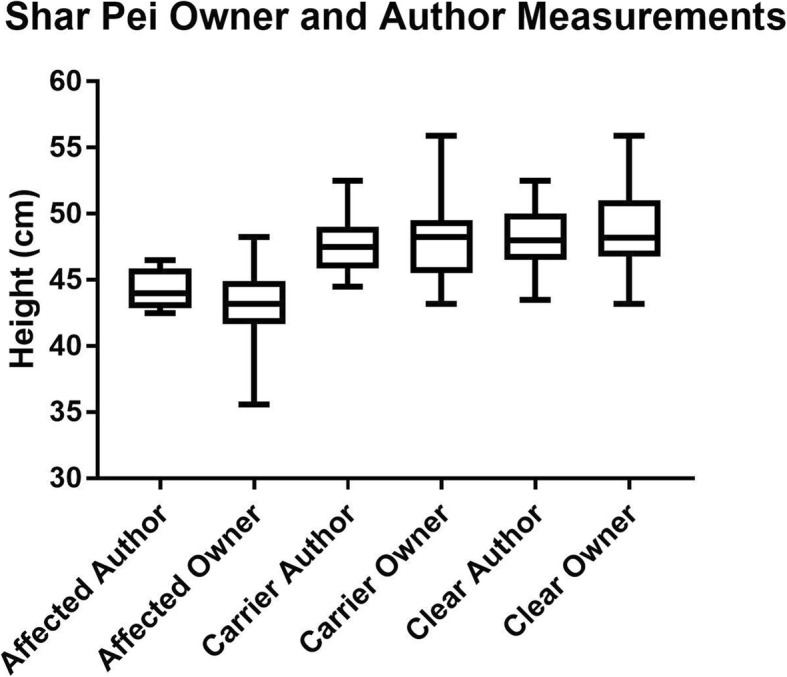
Box and Whiskers plot to show the height at the withers of affected, carrier and clear SPs, stratified by measurer, demonstrating the upper and lower values, the interquartile range and the median value

We assessed the correlation between height measurements of 21 SP that were taken using the stick by owners and the lead author. The correlation was 0.93, indicating a strong correlation between measurements performed between owners and the lead author (*P* = < 0.00001) (Fig. 2, Table 8). There was no statistically significant difference between the mean of these groups (*P* = 0.76).

**Table 8:** Comparison of height measurement results between measurements taken by owners and the lead author using the stick measurement technique

	Mean Height	Number of dogs	SD
Height (author measurement)	47.12 cm	21	2.91
Height (owner measurement)	47.19 cm	21	2.98

#### Height data

The mean height of affected SP (*n* = 9) was 43.32 cm (SD 3.56). The mean height of carrier SP (*n* = 31) was 48.01 cm (SD 3.04). The mean height of clear SP (*n* = 26) was 48.57 cm (SD 2.93) (Fig. 1b).

Using Pearson’s correlation, there was no correlation between height and age for the 60 SP for which the date of birth was available (*P* = 0.43) (Table 9). Linear regression also confirmed this.

**Table 9:** Comparison of height and age of SP

Age group	Number of dogs	Median Height	IQR
Quartile 1	*n* = 15	47.00 cm	3.00 cm
Quartile 2	*n* = 15	48.00 cm	3.53 cm
Quartile 3	*n* = 15	47.00 cm	3.26 cm
Quartile 4	*n* = 15	48.00 cm	6.50 cm

**Fig. 3:**
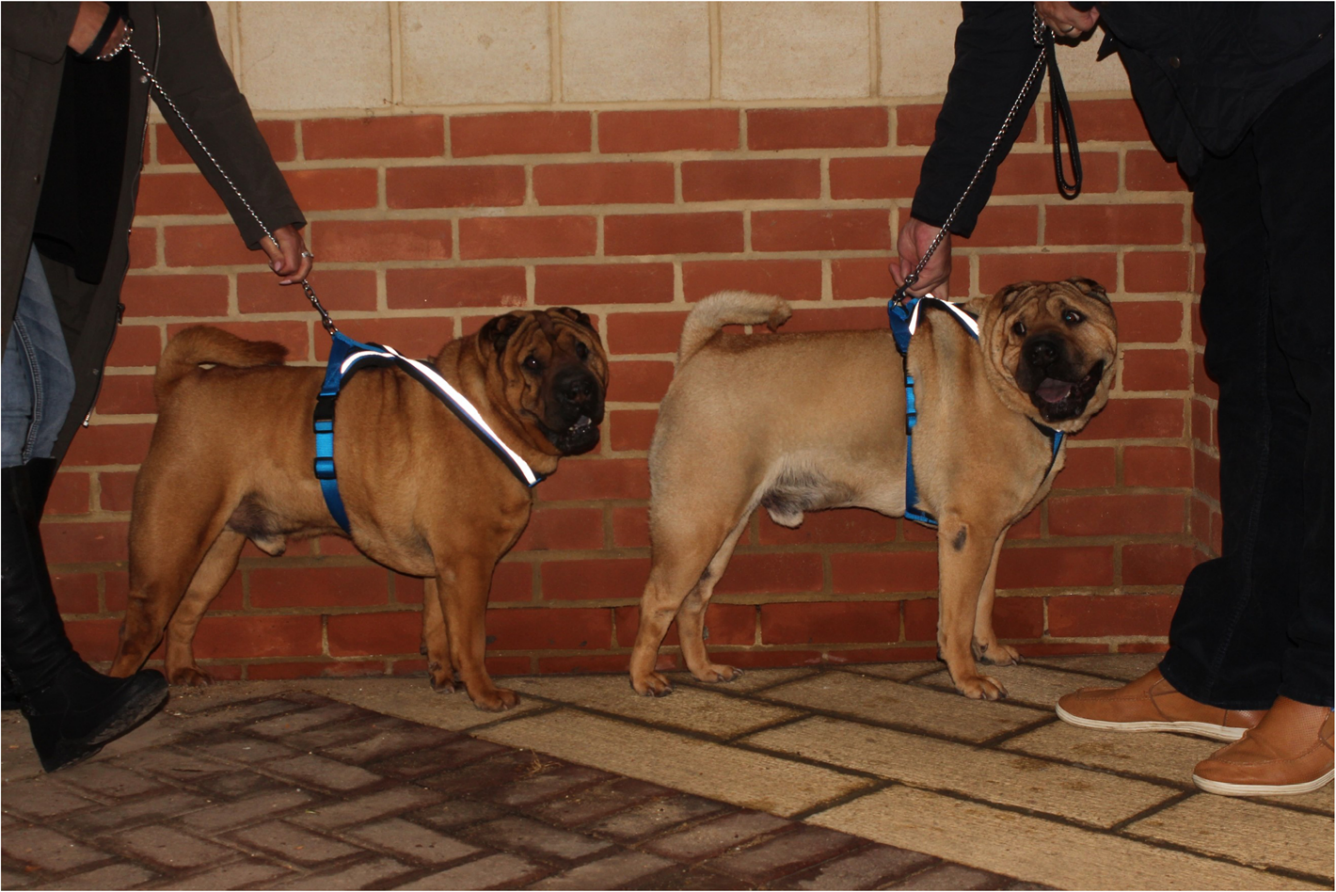
An example of the height difference between two male SP littermates, affected (left), carrier (right)

Similar to the PBGV, we found a statistical association between height and genotype using linear regression (*P* = 0.0004), and the association did not appear linear (*P* = 0.009) (Fig. 1a). Figure 3 shows an example of the height difference between two full sibling male SP, one affected (left) and one a carrier (right).

We generated a variable comprising height using all measures that adjusted for sex, age and method of measurement. With adjustment for these variables, the association between height and genotype increased in significance despite the smaller number of dogs in the analysis (*P* = 0.0001) and showed a linear shape of association (*P*-value for departure from linearity = 0.15) (Fig. 1b). Adjusted mean heights of affected SP (*n* = 9) was 43.96 cm (95% CI 41.88–46.05). The mean height of carrier SP (*n* = 28) was 47.56 cm (95% CI 46.50–48.63). The mean height of clear SP (*n* = 23) was 48.95 cm (95% CI 47.80–50.11). Using a genotypic model with adjustment as above, there was a suggestive difference in height between clear and carrier PBGV (*P* = 0.07) and the difference between clear and affected PBGV was significant (*P* < 0.0001).

## Discussion

Our data show that two breed-specific mutations in *ADAMTS17* in dogs are associated with height as well as POAG/PLL, with affected individuals significantly shorter than clear individuals. Humans with WMLS due to *ADAMTS17* mutations are also shorter than average [14]. It is likely that *ADAMTS17* plays a role in skeletal development in the dog as it does in humans.

Whilst the effect on height found in this study is likely a functional effect of the *ADAMTS17* mutations, this needs to be determined by future studies including functional analyses of the relevant tissue in dogs of different genotype categories. The suggestive effects on height in mutation carriers also needs to be investigated further in larger datasets to confirm or refute a per allele effect. It is also possible that the association with height is due to linkage disequilibrium between an *ADAMTS17* mutation and a nearby gene that influences height, rather than due to a direct effect of *ADAMTS17* itself. Future work could include linkage disequilibrium analysis to confirm or refute this hypothesis.

More variation in height over *ADAMTS17* genotypes was seen in the SP compared to in the PBGV, with far less overlap in the height distribution of affected and carrier/clear dogs. Several factors could potentially be contributing to this difference in effect between breeds. The greater variation in height could be due to the fact that the SP is a much taller breed than the PBGV, with subsequently greater scope for height variation. Therefore, other genetic factors could be impacting on height in the PBGV, subsequently reducing the variance in height of this breed and therefore showing this weaker effect of *ADAMTS17* genotype. Since the PBGV breed has been recently developed from the taller Grand Basset Griffon Vendeen, there has been strong selection pressure on the PBGV for a short stature that has not been placed on the SP. We know that the PBGV segregate an expressed *Fgf4* retrogene insert causing chondrodysplasia, contributing to its short height, height to length ratio < 1 and bowed forelimb conformation [16]. Furthermore, a study by Rimbault et al. [17], looked at the frequencies of six genetic variants which have been shown to be associated with reduced height in various dog breeds. More of these genetic variants were identified, and at greater frequency, in the PBGV than in the SP [17]. Therefore, the presence of multiple other genetic variants which are linked to short height in the PBGV could be modifying the effect of the *ADAMTS17* mutation. Secondly, different mutations are present in *ADAMTS17* in the SP and the PBGV. It is possible that the mutated genes still retain a degree of function, and that this could be different between the two breeds. Further work is necessary to investigate the precise functionality of *ADAMTS17* in these breeds.

Many dog breeds, including the PBGV and SP, are subjected to strong selection pressure to meet specific breed standards. The UK breed standards for the PBGV state that it should stand 34-38 cm high at the withers [18], and the American breed standards specify a height at the withers of 33-38 cm [19]. The SP breed standards (both American and UK) specify that it should stand 46-51 cm high at the withers [20, 21]. Departure from these exact breed standards is heavily penalised in the show ring, so there is likely to be strong selection pressure for genes controlling height.

It is possible that by breeding dogs to meet a specific height criteria, as specified by the breed standard, the frequency of the *ADAMTS17* mutation has been inadvertently increased within the gene pool. It is notable that the *ADAMTS17* mutation is widespread among the PBGV, but has not been reported in the GBGV from which the PBGV breed was developed. The mean heights of affected dogs in our study were at the bottom end or slightly below the heights specified by the UK and American breed standards. However, it is possible that prior to genetic testing, breeders mated affected dogs with taller dogs to try and reduce the height of their offspring. Other dog breeds with described mutations in *ADAMTS* genes are also commonly bred to be of short stature, such as the Basset Hound, the Basset Fauve de Bretagne, the Miniature Bull Terrier, and many other terrier breeds. Indeed, the word “basset” comes from the medieval French for “of low stature” [22]. It could therefore be speculated, given that five different *ADAMTS17* mutations have now been identified in breeds of small stature [3–5, 13], that this locus could be under positive selection.

A significantly higher proportion of PBGV and SP in our study were female than male. Female dogs tend to be shorter than male dogs [23]. Chi-squared analysis did not reveal any significant difference between the male: female ratio between genotypes. Covariable adjustment was performed to ensure that our results were not skewed by the larger proportion of females. The high proportion of female dogs in our study could be due to the proportions of male and female dogs kept by breeders or the proportions presented to dog shows, since the majority of our data were generated at dog shows or by visiting kennels. When the proportion of male and female dogs was grouped by genotype, the ratio of male to female dogs was not significantly different across affected, carrier and clear groups in the PBGV and SP. The male to female ratios were more varied between genotype in the SP, but this could be due to the smaller dataset. Female dogs were found to be shorter than male dogs, so covariate adjustment was performed to adjust for this confounder.

We also analysed the age of the dogs that were presented for our study, as we hypothesised that affected dogs may be presented at a younger age due to the owners being more motivated to enrol them in the study. Only dogs over 12 months old were enrolled in the study to prevent incompletely grown animals from skewing the data. The majority of growth in young dogs occurs between 3 and 6 months, and 90% height is achieved by 9 months [24, 25]. Radiographical analysis of the growth plates in the skeleton indicates that these are closed in the dog by 12 months of age [26]. Affected dogs were on average significantly older than carrier or clear dogs in both breeds. The average age of carrier dogs was also greater than that of dogs clear for the mutation in the PBGV. Covariate adjustment was performed to adjust for this potential confounder. Since DNA tests for the *ADAMTS17* mutations have been available commercially (2015 for the PBGV and 2017 for the SP), breeders have been able to genotype their dogs. We hypothesise that the availability of these tests has enabled breeders to only breed carrier or clear puppies, thus reducing the number of affected puppies being born in recent years.

### Limitations of the study design

Ideally height measurements would have been performed with the examiner blinded to the genotype of the dog, but this was not always possible in this study. When measurements were performed by the authors, information on the genotype was not collected until after the height measurement had been made. However, while clear and carrier animals cannot be distinguished phenotypically, the vast majority of affected animals were severely buphthalmic due to glaucoma, or had had unilateral or bilateral enucleations performed to relieve intraocular pain. These affected animals were therefore clearly identifiable at the time their height was measured. When height measurements were performed by the owners, they were already aware of the genotype of their dog.

Many of the measurements were performed by the owners of the dogs, rather than by the authors, and there was variation in the different measuring techniques used. Variations in technique and between measurers will have introduced a degree of variability into the data set, although we did adjust for this in our analyses to some extent by generating a ‘measurement’ covariate that we included in our regression models. However, by allowing data to be collected by owners we were able to obtain a far larger dataset, including data from animals worldwide, which would otherwise not have been possible. Written instructions with diagrams were provided to all owners who were asked to measure their dog’s height to help minimise individual variation in measuring technique. We expected that the variability introduced by having multiple individuals involved in data collection would be offset by the increased amount of data available. Analysis of the data provided by owners compared with that provided by authors did show that the owner measurements had a greater variability. However, comparison between measurements taken by owners and the author at a show showed a strong correlation between measurement techniques.

The laser measurement technique measured dogs as “shorter” than the commercially available measuring stick. We found that when the laser technique was used, the dogs tended to crouch as the ruler was rested on their back. Covariate adjustment was therefore used to ensure that this difference did not affect the height analysis while not losing any power from the study.

The mean age of the affected dogs available to the study was higher than that of the mean age of the population. This may be because fewer affected dogs are likely to have been bred since the DNA tests became available. The increased average age of carriers compared to clear dogs in the PBGV indicates that there is also a trend for breeders to select clear dogs over carriers for breeding purposes. Covariate adjustment was used to adjust for the confounder of age. This increased both the strength and linearity of the association between height and *ADAMTS17* genotype, as older dogs were slightly taller, but affected dogs were both older and significantly shorter.

## Conclusions

Two independent mutations causing POAG in two independent breeds are also associated with short stature in these breeds. We suspect that selection for short stature has inadvertently led to an increase in mutation frequency within each breed and thus increased prevalence of POAG.

## Methods

### Study design and population

PBGV and SP that had been genotyped for their breed-specific *ADAMTS17* mutation were invited to take part in the study. (These DNA tests are commercially available.) The dogs were classified as affected, carrier or clear. The height of each dog was measured at the withers. Height measurements were performed by the authors (EJ, CM, JO and DG) at different locations across the UK between December 2016 and March 2018; by officials at breed shows; or by the owners of the dogs. These sessions took place at various events; including dog shows; breed information days; and visits to kennels. The measuring sessions were arranged by contacting owners who had purchased DNA tests from the Animal Health Trust directly, and promoted via breed club websites, social media, and word of mouth. All dogs over the age of 1 year that were volunteered for measurement were accepted, regardless of age, sex, or Kennel Club registration status. Dogs less than 1 year of age at the time of measurement were excluded from the analysis. For each dog, data were collected on sex and age at time of measurement. If data on sex and age were not supplied by the owners, these data were obtained from Kennel Club online records if available. All dogs were pets and measurement was only performed after informed owner consent. All experiments were approved by the Animal Health Trust’s Research and Ethical Approval Committee (Ref. 44-2017E).

### Measurement procedure

**Fig. 4:**
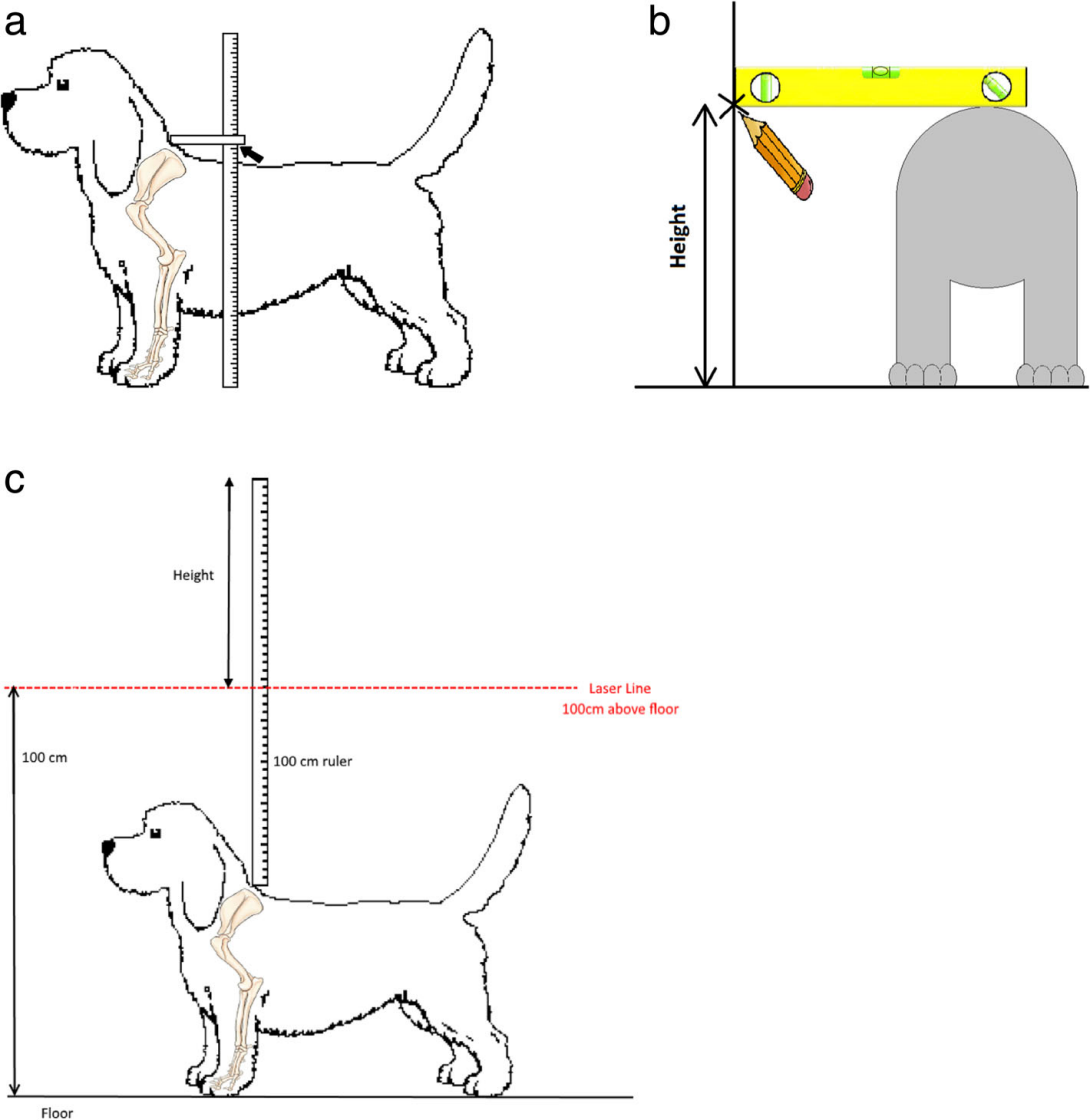
(Additional files 1 and 2). Methods of measuring the height of dogs. **a**. Use of a commercially available measuring stick to measure the height at the withers. **b**. Use of a wall as an aid for measurement of the height at the withers, when a commercially available measuring stick is not available. **c**. Use of the laser method for measuring the height of a dog at the withers

Each dog was stood square on a flat level surface and held by an assistant, and the top of the withers was palpated. A ruler held parallel with the floor with the aid of a spirit level was lowered onto the withers and held in contact with the skin. The distance between the base of this ruler and the floor was then measured and recorded as the dog’s height. The measurement was done either directly with the aid of a commercially available device (http://www.dogmeasuringsticks.co.uk/) (Fig. 4a, Additional files 1 and 2), or indirectly by marking a wall at the level of the withers, then measuring the distance between the mark and the floor (Fig. 4b, Additional files 1 and 2).

These measurements were either done by the authors, by officials at a show, or by the owners at home and the results emailed to the authors. Written instructions with diagrams were provided to all owners who were asked to measure their dog’s height to help minimise individual variation in measuring technique (Additional file 3). A subset of SP at a show were measured both by the author (EJ) and by the owner to compare the accuracy of owner measurements. The owners were blinded to the result of the measurement taken by the author.

Alternatively, a ruler held vertically with “0” at the top was lowered onto the withers and held in contact with the skin. A laser pointer was set to dissect the ruler at 100 cm from the floor. The point at which the laser crossed the ruler was recorded as the dog’s height (Fig. 4c, Additional files 1 and 2).

### Statistical analyses

Age at measurement (in years) was calculated by generating an age variable = (date of measurement – date of birth)/365.25. If the dog had been measured more than once, the date of the first measurement with the stick was used to calculate the age. In both the PBGV and SP age was not normally distributed (Shapiro-Wilk test, SPSS Statistics, IBM, NY, USA). For non-parametric data, a Kruskal-Wallis one-way ANOVA (SPSS Statistics, IBM, NY, USA) was used to assess for significant difference in age between genotypes, with *P* < 0.05 considered to be significant. Comparison between genotype groups (clear vs. affected, clear vs. carrier, and carrier vs. clear) was performed using Mann-Whitney U tests, employing Bonferroni’s correction with *P* < 0.017 considered to be significant (SPSS Statistics, IBM, NY, USA).

Chi-squared analysis with Yates’ correction (Microsoft Excel, Microsoft Office, LA, USA) was used to assess whether the male: female ratio of animals presented varied significantly from the 50:50 ratio expected, with *P* < 0.05 considered to be significant. Chi-squared analysis was used to assess for any difference between the male:female ratio between genotypes, using the overall male:female ratio as the expected ratio. Comparison between the height of male and female dogs was employed for all dogs and for each genotype using Mann-Whitney U tests, with *P* < 0.05 considered to be significant (SPSS Statistics, IBM, NY, USA).

For height data, the measurement taken by the author using the measuring stick was used where available. If two or more stick measurements were available the mean of both measurements was used. If a measurement taken by the author was not available, then a measurement taken by the owner, at a show or by the laser method was used. The Shapiro-Wilk test (SPSS Statistics, IBM, NY, USA) was used to assess height data for normality, with *P* < 0.05 considered to be significant. Mann-Whitney U tests (SPSS Statistics, IBM, NY, USA) were used to compare height data between male and female dogs for the whole dataset and for each genotype, with *P* < 0.05 considered to be significant. Kruskal-Wallis one-way ANOVA (SPSS Statistics, IBM, NY, USA) was used to assess for significant difference in height between genotypes, with *P* < 0.05 considered to be significant. Comparison between genotype groups (clear vs. affected, clear vs. carrier, and carrier vs. clear) was performed using Mann-Whitney U tests, employing Bonferroni’s correction with *P* < 0.017 considered to be significant (SPSS Statistics, IBM, NY, USA).

The effect of age on height was analysed using Pearson correlation (SPSS Statistics, IBM, NY, USA), with *P* < 0.05 considered to be significant. We also analysed this using linear regression coding age into quartiles.

For all analyses with all data a ‘measure’ variable was generated for use as a covariate in the regression analyses to adjust for the variation in measurement method. For PBGV this was made up of four values: 1 = stick; 2 = laser (if stick was not available); 3 = owner (if stick and laser were not available); 4 = show (if stick, laser and owner were not available). For SP this was: 1 = stick; 2 = owner (if stick was not available).

The correlation between measurement methods and between author and owner-generated data was assessed using Pearson’s pairwise correlation (STATA 10.0, College Station, TX, USA), and tested for a significant difference between the means between these groups using a paired t-test (Microsoft Excel, Microsoft Office, LA, USA). We used linear regression (per allele and genotypic models) to assess the association between the PBGV and SP *ADAMTS17* mutations and height as a continuous variable (unadjusted analysis – measurement method as a covariate only); and to assess the association between *ADAMTS17* mutations and height using a model adjusted for measurement method, age (continuous variable) and sex (binary variable). (Age was assessed for departure from linearity by comparing a model containing age as a continuous variable to a model containing age as an indicator variable using the likelihood ratio test. As there was no statistical difference between these two models, age was kept as a continuous variable.) We used linear regression and likelihood ratio tests to compare a general model with a linear (per allele) model to assess the shape of the association between *ADAMTS17* mutations and height, using STATA 10.0 (College Station, TX, USA).

## Additional files

Additional file 1: Raw data from PBGV measurements. (XLSX 47 kb)

Additional file 2: Raw data from SP measurements. (XLSX 18 kb)

Additional file 3: Written instructions for the measurement of height provided to owners. (DOCX 252 kb)

## Abbreviations

*ADAMTS*: A Disintegrin and Metalloproteinase with Thrombospondin motifs
*ADAMTS17*: A Disintegrin-like and Metalloproteinase with Thrombospondin type 1 motif, 17
CI: Confidence Interval
DNA: Deoxyribonucleic Acid
ECM: Extracellular Matrix
IOP: Intraocular Pressure
IQR: Interquartile Range
PBGV: Petit Basset Griffon Vendeen
PCAG: Primary Closed Angle Glaucoma
PLL: Primary Lens Luxation
POAG: Primary Open Angle Glaucoma
RGC: Retinal Ganglion Cells
SD: Standard Deviation
SP: Shar Pei
UK: United Kingdom
USA: United States of America
WGS: Whole Genome Sequence
WMLS: Weill-Marchesani-like Syndrome
WMS: Weill-Marchesani Syndrome
