# Supplementary material for: Glaucoma-causing ADAMTS17 mutations are also reproducibly associated with height in two domestic dog breeds: selection for short stature may have contributed to increased prevalence of glaucoma
Source: Canine Genet Epidemiol. 2019 May 17;6:5. doi: 10.1186/s40575-019-0071-6 (PMC6524303; doi:10.1186/s40575-019-0071-6)
Supplement: Supplementary file 3 — Written instructions for the measurement of height provided to owners. (DOCX 252 kb) [file 40575_2019_71_MOESM3_ESM.docx]

Appendix A: Owner instructions for measuring height.

How to measure your dog’s height.

Equipment:

- Spirit level (or you can use an app on your phone – see below!)
- Flat, non-slip surface next to a blank wall or doorframe
- Pencil
- Measuring tape or long ruler
- Assistant

If you don’t have a spirit level, you can download a free app so you can use your phone instead. Search for “i Handy Level” on an iPhone, or “Bubble level” on an Android. You might need to tape a ruler to your phone to make it long enough to use for measuring. Make sure the ruler is level with the edge of the phone.


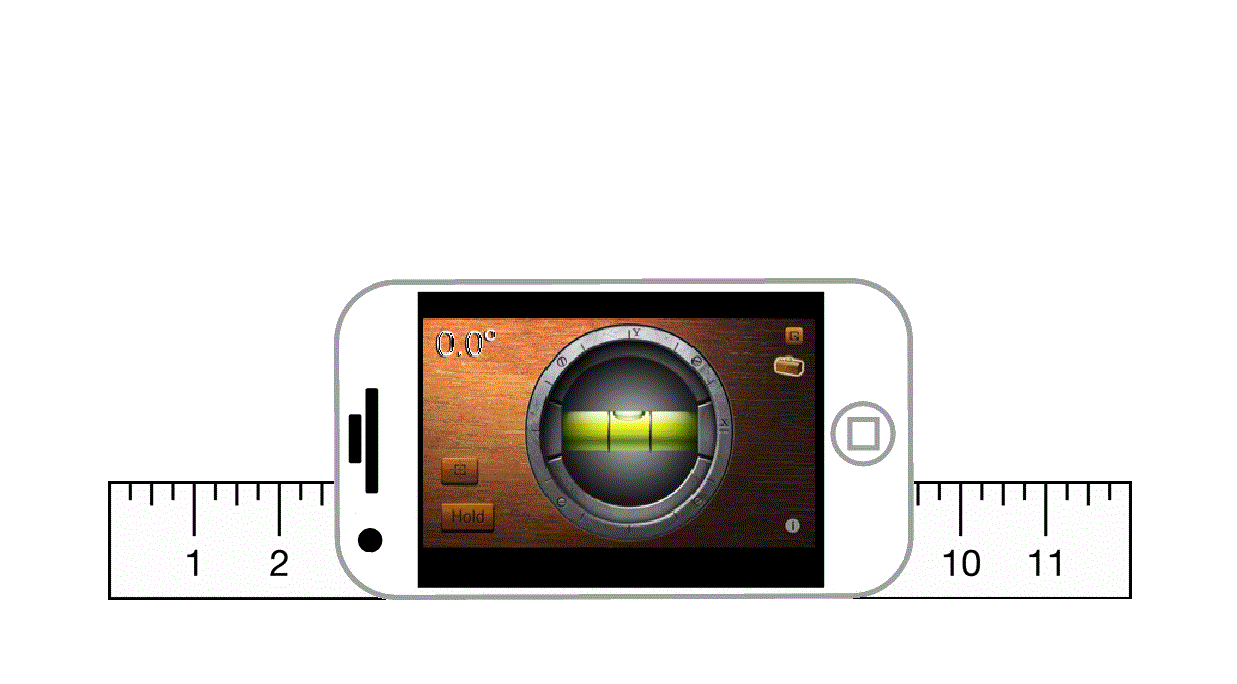


Ask your helper to get your dog to stand square on a flat surface next to the wall. This is the same position used when showing. Your dog should not be crouched or stretched, and his/her head should be in a natural position.


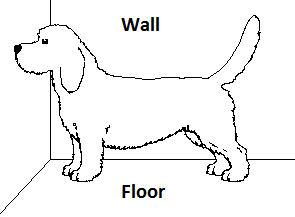


Place the spirit level across the highest point of the dog’s withers (shoulder blades). Apply gentle pressure so that the spirit level is touching the skin and not resting on the hair (the hair on some dogs can be quite thick!)

One end of the spirit level should touch the wall. The bubble should be in the middle of the level indicator.


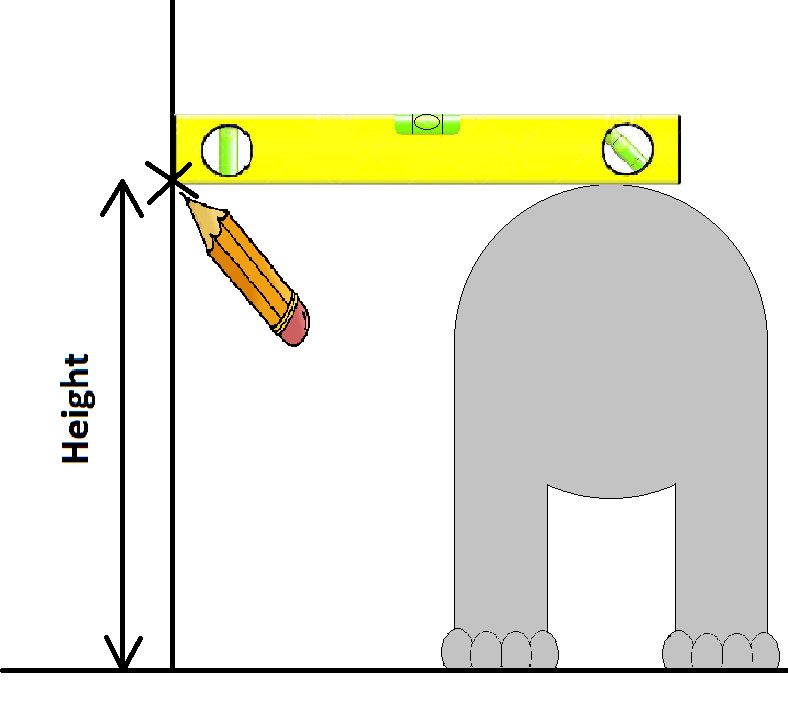

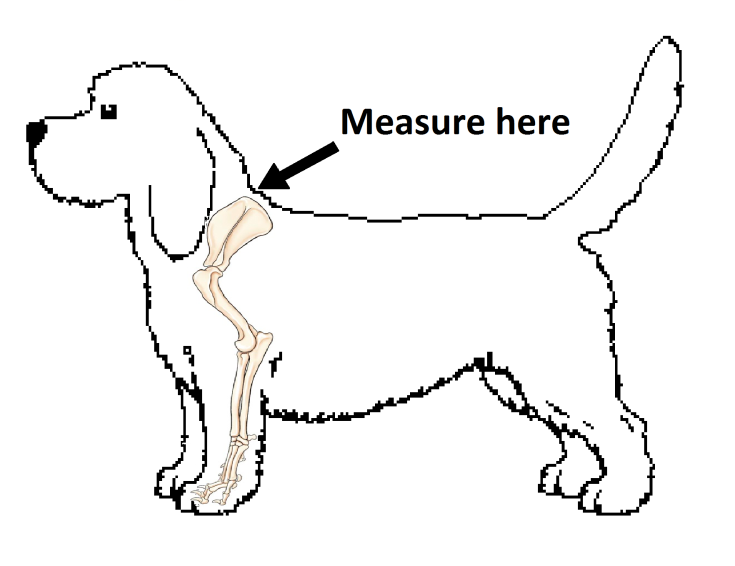


Use the pencil to mark the wall underneath where the level touches the wall.

Measure the distance from the mark on the wall to the floor. This distance is the height of your dog.

This method is more accurate than simply holding a measuring tape next to the dog, because if the tape is not perfectly straight you will get errors.
